# A trans-activating transduction peptide fused nanobody targeting viral nonstructural protein NS4B inhibits bovine viral diarrhea virus replication

**DOI:** 10.3389/fimmu.2025.1712683

**Published:** 2025-12-12

**Authors:** Bao Zhao, Zehao Lv, Wenqi Fan, Ying Zhang, Shuang Wei, Junyang Fang, Ni Huang, Xiwen Chen, Lizhen Wang, Xuefeng Qi

**Affiliations:** 1 College of Veterinary Medicine, Northwest A&F University, Yangling, Shaanxi, China; 2 Key Laboratory of Ruminant Disease Prevention and Control (West), Ministry of Agriculture and Rural Affairs, Xi’an, China; 3 Fisheries Research and Technology Extension Center of Shaanxi , Xi’an, China; 4 Animal Disease Prevention and Control and Healthy Breeding Engineering Technology Research Center, Mianyang Normal University, Mianyang, Sichuan, China

**Keywords:** BVDV, NS4B, nanobody, trans-activating transduction, antiviral agents

## Abstract

**Introduction:**

Bovine viral diarrhea virus (BVDV) is a major pathogen in cattle globally. Nanobodies (Nbs) possess attractive therapeutic properties, but their efficacy against intracellular targets like BVDV is hindered by poor membrane permeability.

**Methods:**

A nanobody (Nb91) targeting the BVDV nonstructural protein NS4B was isolated. Nb91 was intracellularly expressed to assess its antiviral effect. Furthermore, Nb91 was fused to the cell-penetrating TAT peptide to generate TAT-Nb91. The internalization efficiency of TAT-Nb91 and its inhibitory effects against both cytopathogenic (CP) and noncytopathogenic (NCP) BVDV biotypes were evaluated in MDBK cells and in bovine endothelial (Bend) cells, the primary *in vivo* targets of BVDV. The epitope of Nb91 on NS4B was mapped.

**Results:**

Intracellularly expressed Nb91 potently suppressed BVDV replication in MDBK cells (~100% inhibition). TAT-Nb91 was effectively internalized in a dose- and time-dependent manner. It inhibited CP and NCP BVDV replication in MDBK cells by approximately 70% and 40%, respectively, and achieved over 50% inhibition for both biotypes in Bend cells. Epitope mapping identified NS4B amino acids 328-347 as the critical binding region for Nb91.

**Discussion:**

These findings demonstrate that a cell-penetrating nanobody targeting the conserved viral protein NS4B can effectively inhibit BVDV replication across relevant cell types and biotypes. This establishes TAT-fused nanobodies as a promising new therapeutic strategy against BVDV.

## 1 Introduction

Bovine viral diarrhea virus (BVDV) is a significant pathogen primarily affecting cattle, with a broad host range that includes various domestic and wild species such as sheep, goats, deer, camelids, pigs, and other wildlife (1, 2). Infection with BVDV leads to diverse clinical manifestations including immunosuppression, enteritis, respiratory disorders, and reproductive failures in bovines (3). Currently, effective strategies for controlling or treating BVDV infection remain limited, largely due to the complex pathogenesis of the disease (4). Therefore, developing efficient therapeutic targets for combating BVDV infections are imperative.

BVDV is a positive-sense RNA virus classified within the genus *Pestivirus* of the family *Flaviviridae*. Its approximately 12.3 kb RNA genome contains a single open reading frame flanked by 5′ and 3′ untranslated regions (UTRs), and encodes a polyprotein that is subsequently cleaved by viral and host proteases into both structural proteins—including the capsid protein and three envelope glycoproteins (E^rns^, E_1_, E_2_)—as well as nonstructural proteins (NSPs) such as N^pro^, P7, NS2/3, NS4A, NS4B, NS5A, and NS5B (5). Among these, NS4B is essential for viral replication and has been implicated in subverting the host’s innate antiviral response, particularly the RNA-sensing pathways (6). Additional roles of NS4B in viral pathogenesis and the induction of autophagosomes have also been proposed (7), suggesting it an attractive target for antiviral drug development. Although RNA viruses exhibit high genetic variability, especially in the neutralizing epitopes of structural proteins due to immune pressure, whereas nonstructural proteins like BVDV NS4B, which function in cytoplasmic replication complexes, are typically more conserved (8).

Nanobodies (Nbs), the smallest antigen-binding fragments derived from camelid heavy-chain antibodies (VHH), possess several advantageous properties such as small size (≈15 kDa), high stability, strong binding affinity, and efficient tissue penetration (9–11). These attributes render them suitable for both diagnostic and therapeutic applications. Accumulating evidence supports their potential in antiviral therapy. For instance, the trivalent nanobody ALX-0171 inhibits respiratory syncytial virus (RSV) replication by targeting viral fusion proteins (12, 13). Similarly, nanobody Nb25 neutralizes duck hepatitis A virus by binding a conserved B-cell epitope (14), and Nb6 targets PRRSV Nsp9 to attenuate viral replication (15). Although an intracellularly expressed nanobody targeting BVDV NS5B was shown to potently inhibit viral replication (15), the potential of other key nonstructural proteins, such as NS4B, as therapeutic targets for nanobodies remains unexplored.

As the inefficient permeability of the cell membrane to macromolecules limits the broader application of nanobodies in therapeutics, it is pertinent to establish an efficient, safe, and nontoxic delivery system to greatly improve the applicability of nanobodies. Cell-penetrating peptides (CPPs), such as the trans-activating transcriptional activator (TAT) from HIV, are short peptides (5–30 amino acids) capable of facilitating the delivery of bioactive cargoes into cells via direct translocation or endocytosis, without significant cytotoxicity (16, 17). They have been employed to transport diverse molecules, including siRNA, DNA, proteins, viral particles, and nanoparticles (18).

In this study, a novel nanobody specific to BVDV NS4B, designated Nb91, was identified and expressed in a prokaryotic system. Furthermore, a fusion protein combining TAT peptide with Nb91 was produced in *E. coli*. We demonstrate that TAT-Nb91 efficiently enters Madin-Darby bovine kidney (MDBK) cells and importantly, TAT-Nb91 can effectively inhibits the replication of both cytopathogenic (CP) and noncytopathogenic (NCP) biotypes of BVDV *in vitro*.

## 2 Materials and methods

### 2.1 Cells and viruses

HEK-293T and Bovine endometrial epithelial (Bend) cells were procured from the China Center for Type Culture Collection (CCTCC, Beijing), and MDBK cell lines were procured from the American Type Culture Collection (ATCC CCL-22). Cells were cultured in Dulbecco’s modified Eagle’s medium (DMEM; Life Technologies, USA) containing 10% fetal bovine serum (Gibco), 100 IU/mL penicillin, and 10 μg/mL streptomycin (Hyclone) at 37 °C in a 5% CO_2_ atmosphere.

The BVDV field strain HJ-1 (genotype 1b, cytopathogenic; GenBank: JX065783) was originally isolated from a deceased Holstein cow showing signs of mucosal disease. This strain was selected owing to its pronounced cytopathic effect (CPE) in MDBK cells (4). The noncytopathogenic (NCP) BVDV strain New York 1 (genotype 1b; GenBank: FJ387232) was obtained from the China Veterinary Culture Collection Center. Both virus stocks were prepared by three freeze-thaw cycles of infected cell cultures (4). Viral titers were determined via endpoint titration and calculated as TCID_50_ using the Reed-Muench method.

### 2.2 Expression and purification of recombinant NS4B protein

The NS4B gene (GenBank: KJ608492.1) was amplified by PCR assay from BVDV cDNA and cloned into the pET-28a vector (Novagen, Darmstadt, Germany). The primers used for PCR amplification are listed in **Table 1**. The resulting recombinant plasmid, pET28a-NS4B, was extracted using the Plasmid AutoExtraction kit-DeepWell Plate (Kangma-Healthcode, Shanghai, China), and was transformed into E. coli Transetta (DE3) cells (Transgene Biotech, Beijing, China). Protein expression was induced with 1 mM IPTG at 25 °C for 18 h. Bacterial pellets were resuspended in lysis buffer (20 mM Tris-HCl, pH 8.0, 300 mM NaCl, 10 mM imidazole, 1 mM DTT), lysed by sonication, and centrifuged. The supernatant was subjected to Ni-NTA affinity chromatography (Qiagen, Hilden, Germany), and the eluted protein was further purified by size-exclusion chromatography on a Superdex 200 column (GE Healthcare Life Science, Pittsburgh, USA). Purified NS4B-His protein was analyzed by SDS-PAGE and Western blot.

**Table 1:** Primers used for construction of plasmid pET28a-NS4B, pET-21b-TAT-Nbs and pET-21b-Nbs.

Primer name	Sequence (5’-3’)	Purpose
NS4B-F	GCGGATCCGCGTCGGGTGACGTGG	NS4B
NS4B-R	CGCTCGAGCAGGTTCCTTATTTTCC	
TAT-Linker-Nb-F1	CCAAGCTTTACGGTCGTAAGAAACGTCGCCAGCGTCGCCGTGGAGGCGGTGGCTCGGGC	pET-21b-TAT-Nbs
TAT-Linker-Nb-F2	GGAGGCGGTGGCTCGGGCGGTGGCGGCTCGGGTGGCGGTGGTTCTCAGGTGCAGCTGCAGGAG	
VHH-F	CCAAGCTTCAGGTGCAGCTGCAGGAGTCT	pET-21b-Nbs
VHH-R	CCGCTCGAGTGAGGAGACGGTGACCTG	

### 2.3 Camel immunization and nanobody library construction

A 4-year-old male Alashan Bactrian camel was immunized subcutaneously six times at two-week intervals with 5 mg of recombinant NS4B protein emulsified in Freund’s adjuvant (19). Serum antibody titers were evaluated by indirect ELISA using NS4B-His as the coating antigen. After the final immunization, peripheral blood mononuclear cells (PBMCs) were isolated from 250 mL of blood. Total RNA was extracted, cDNA was synthesized, and VHH fragments were amplified by nested PCR. The amplified products were ligated into the phagemid vector pCANTAB 5E (GE Healthcare Life Science, Pittsburgh, USA) to construct a bimodal camel heavy chain antibody variable region library.

### 2.4 Screening and identification of NS4B-specific nanobodies

Phage display biopanning was carried out over three rounds against immobilized NS4B (19). Enrichment of specific binders was monitored by using anti-M13/HRP conjugate ELISA together with phage titration. A total of 121 individual clones were randomly selected, expressed, and screened for NS4B binding using periplasmic extracts and anti-E-tag iELISA. Positive clones were sequenced and clustered based on CDR3 homology. High-affinity nanobodies were chosen for further analysis.

### 2.5 Production of recombinant nanobodies

Genes encoding selected nanobodies (Nb91, Nb5) and TAT fusion constructs were cloned into the pET-21b(+) plasmid (Novagen, Darmstadt, Germany), containing the sequences for a 6-histidine (6×His) tag downstream of the gene insertion site. The recombinant plasmids, named pET21b-Nb91 and pET21b-Nb5, were transformed into *E. coli* BL21(DE3), and protein expression was induced with 1 mM isopropyl *β*-D-1-thiogalactopyranoside (IPTG) at 37 °C for 6 h. Recombinant proteins in inclusion bodies were dissolved in 8 M urea for denaturation and then purified using Ni-NTA resin (Roche, Mannheim, Germany) according to the manufacturer’s instructions. The denatured proteins were refolded by rapid dilution in base refolding buffer (880 mM L-arginine, 55 mM Tris, 21mM NaCl, 0.88mM KCl, pH 8.2) with 10 mM EDTA, 150mM reduced glutathione, and 15 mM oxidized glutathione. For the subsequent cell experiments, the refolded proteins were dialyzed into 0.01 M phosphate-buffered saline (PBS), analyzed by SDS-PAGE and Western blotting, and stored at -80 °C until use.

### 2.6 Cellular internalization assay

MDBK cells were seeded in 24-well plates at a density of 1 × 10^5^ cells per well and treated with serum-free culture medium containing TAT-Nb91, or Nb91 at different concentrations for 0, 1, 3, 5, and 10 h. Then, the cells were washed three times and internalized proteins were detected by Western blot, immunofluorescence, and flow cytometry.

### 2.7 Cell viability assay

The viability of MDBK cells was assessed using a Cell Counting Kit-8 (CCK-8) assay as previously described (4). Briefly, cells were seeded in 96-well plates 1 × 10^4^/well), and absorbance was measured daily for one week to construct proliferation curves.

### 2.8 Antiviral activity assay

MDBK cells were infected with BVDV at an MOI of 1 and treated with TAT-Nb91, Nb91, TAT-Nb5, or Nb5, respectively. At 12, 24, 36, and 48 hours post incubation, supernatants and cells were harvested for viral titration, Western blot, and qPCR analysis.

### 2.9 Indirect enzyme-linked immunosorbent assay

Binding activity of TAT-Nb91 was determined by indirect enzyme-linked immunosorbent assay (iELISA) (1). Briefly, the 96-well microplates were coated with NS4B protein (400 ng/well), incubated with TAT-Nb91, and detected using anti-camel serum (diluted to 1:2,000) and horseradish peroxidase (HRP)-conjugated goat anti-mouse IgG (1:5,000, Jackson ImmunoResearch Laboratories, West Grove, PA, USA). Absorbance was measured at 450 nm using an automated ELISA plate reader (Bio-Rad, Hercules, CA, USA).

### 2.10 Immunofluorescence assay

Immunofluorescence assay (IFA) was performed as previously described (4), with the following modifications. Briefly, the cells were fixed with 4% paraformaldehyde (Sigma-Aldrich), permeabilized with 0.2% Triton X-100 (Sigma-Aldrich), blocked with 1% bovine serum albumin (BSA), and incubated with mouse anti-His antibody (1:1,000; Tiangen, Beijing, China) followed by Incubations with Alexa Fluor 488- or Alexa Fluor 555-conjugated goat anti-mouse IgG (HL) (1:500; ThermoFisher Scientific, USA). Nuclei were stained with 4’,6-diamidino-2-phenylindole (DAPI), and the stained cells were observed under a confocal microscope (AF6000; Leica, Wetzlar, Germany).

### 2.11 Real-time quantitative PCR

Total RNA was extracted with TRIzol reagent (Invitrogen, CA, USA), reverse transcription reactions were performed using a PrimeScript RT master mix kit (TaKaRa, Dalian, China). The qPCR was performed using a StepOne Plus Real-Time PCR System (Applied Biosystems; Thermo Fisher Scientific Inc.) and FastStart Universal SYBR Green Master (Roche Diagnostics GmbH) in a 10 µL reaction volume. Reaction conditions were 95°C for 10 min, 95°C for 15 s, and 60°C for 1 min with 40 cycles. Relative expression of the NS4B gene was calculated using the 2−ΔΔCT method by relative qPCR, with Glyceraldehyde-3-phosphate dehydrogenase (GAPDH) as the internal control. The sequences of the primers used for qPCR amplification are listed in **Table 1**.

### 2.12 Virus titration

Virus progeny was detected by titration as previously described (5). MDBK cells seeded on 96-well plates were infected with serial dilutions of BVDV, and the 50% tissue culture infective dose (TCID_50_) was calculated using the Reed-Muench method.

### 2.13 Western blot analysis

Western blotting was performed as previously described (4), with the following modifications. Briefly, cells were harvested and lysed, and the cellular proteins were separated by SDS-PAGE, transferred to PVDF membranes, and probed with antibodies against His-tag (1:2,000), NS4B (1:5,000), or β-actin (1:5,000; Sigma-Aldrich, St. Louis, MO, USA). Blots were detected using HRP-conjugated secondary antibodies at a 1:2,000 dilution and ECL chemiluminescent detection system (Pierce, Rockford, IL, USA).

### 2.14 Pulldown assay

Pulldown assay was carried out as described previously (4). MDBK cells were infected with BVDV field strain HJ-1 at an MOI of 1 for 48 h, washed with PBS three times, and then lysed with lysis buffer (50 mM Tris-HCl, 150 mM NaCl, 0.2mM EDTA, 2mM EGTA, 10% glycerin, 0.5% Triton X-100) containing a proteinase inhibitor cocktail (Roche, Mannheim, Germany). The cell lysates were collected by centrifugation at 14,000 × g for 15min at 4 °C and mixed with TAT-Nb91 or TAT-Nb5, and then the mixtures were incubated with PureProteome nickel magnetic beads (Millipore, USA). Antigen bound beads were washed three times with wash buffer (pH 8.0). The bound proteins were eluted with elution buffer (pH 8.0) and analyzed by Western blotting.

### 2.15 Statistical analysis

Data are presented as mean ± standard error of the mean (SEM) from triplicate experiments. Statistical significance was determined using two-way ANOVA with Bonferroni’s *post hoc* test or Student’s *t*-test. A *p*-value < 0.05 was considered significant. All analyses were performed using GraphPad Prism 8.

## 3 Results

### 3.1 Antigen preparation and construction of a VHH library from an NS4B-immunized camel

**Figure 1:**
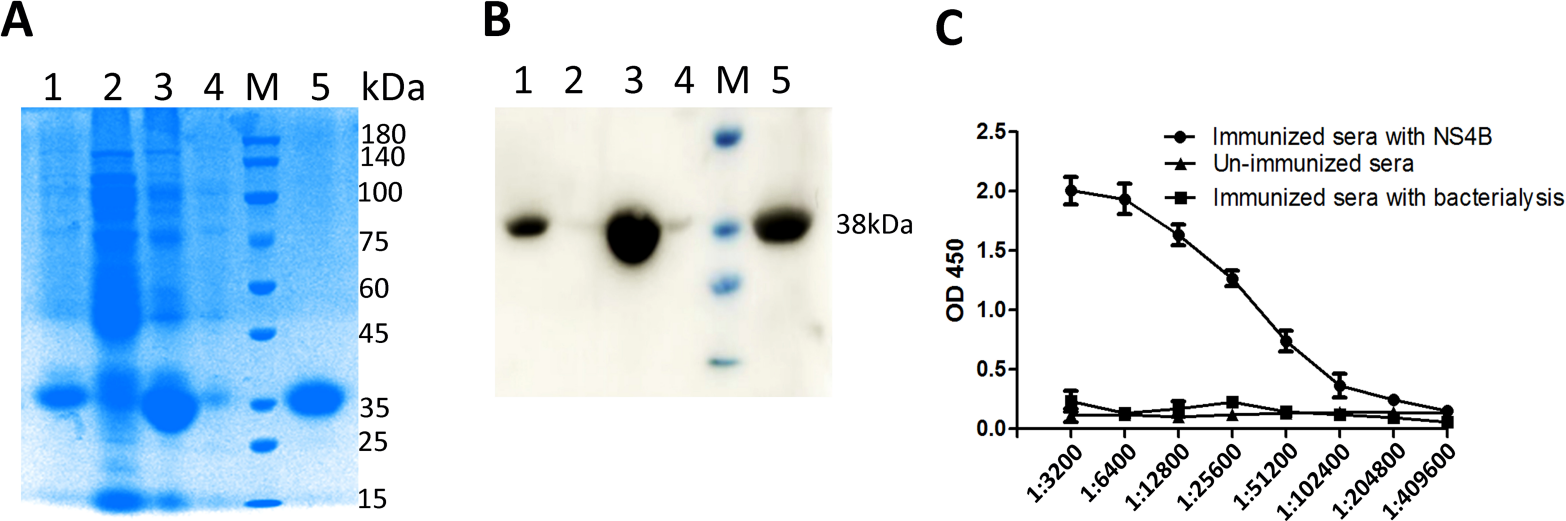
Expression, purification, and identification of BVDV NS4B recombinant protein and detection of serum anti-NS4B antibody titer. The expression and purification of NS4B recombinant protein were analyzed by SDS-PAGE **(A)** and Western blotting **(B)**. Lane 1: inclusion body in precipitate after sonication; Lane 2: soluble protein in supernatant after sonication; Lane 3: induction with 0.5 mM IPTG; Lane 4: uninduced expression of bacterial liquid (whole bacteria); lane 5: purified NS4B protein; M: molecular weight markers, size indicated in kDa. Determination of serum antibody titers against NS4B after immunization by iELISA **(C)**.

The NS4B recombinant protein was expressed in *E. coli* Transetta (DE3) cells in the form of inclusion bodies. The protein was subsequently purified using Ni-NTA affinity chromatography followed by gel filtration on a Superdex 200 column. SDS-PAGE analysis showed that the molecular weight of the NS4B-His recombinant protein was approximately 38 kDa (**Figure 1A**). Western blot analysis using an anti-His monoclonal antibody further verified the identity of the purified protein, showing a clear band at the expected size (**Figure 1B**). The purified NS4B protein was used both for camel immunization and as a coating antigen in subsequent nanobody screening. Serum samples collected from the Alashan Bactrian camel before and after immunization were analyzed by iELISA to evaluate the immune response. The anti-NS4B serum antibody titer increased to 1:102,400 following the final immunization (**Figure 1C**). A phage-displayed VHH library was constructed using peripheral blood mononuclear cells (PBMCs) isolated from the immunized camel, yielding approximately 3.5 × 10^8^ individual colonies. Colony PCR indicated that 98% of the colonies contained inserts of the correct size for VHH genes. Sequencing of 100 randomly selected clones confirmed that each contained a unique VHH sequence, indicating high library diversity and quality.

### 3.2 Isolation and identification of NS4B-specific nanobodies

**Figure 2:**
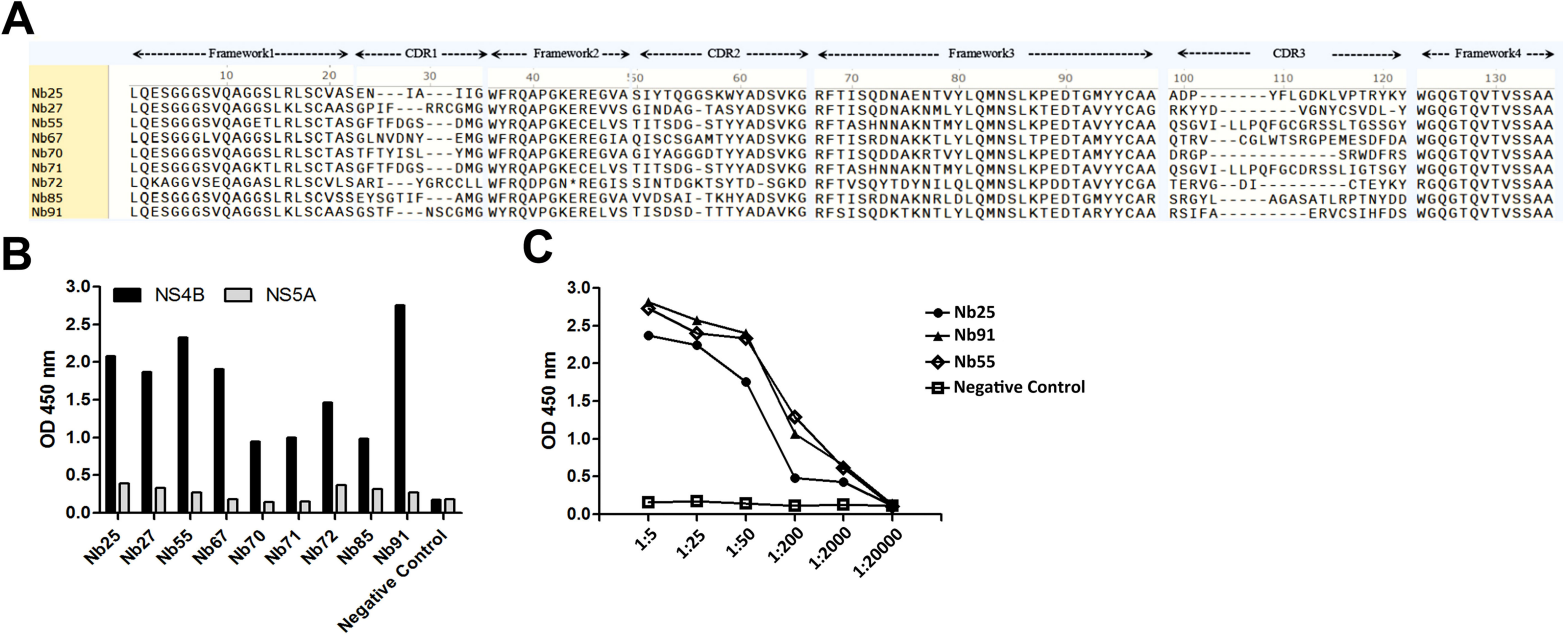
Isolation and identification of NS4B-specific nanobodies. Alignment of amino acid sequences of NS4B-specific nanobodies **(A)**. Detection of the binding of the nine unique nanobodies against NS4B using iELISA **(B)**; BVDV-NS5A-His protein was used as a control. Titration of binding the three screened nanobodies with the NS4B protein by iELISA **(C)**; Nb5 was used as a negative antibody control, 4 µg/ml NS4B protein was the coating antigen; different dilutions of nanobodies were added; E-tag as a primary antibody.

Biopanning against immobilized NS4B-His protein was performed to enrich NS4B-specific phage particles over three rounds (**Table 2**). A total of 120 clones from the third round of panning were subjected to iELISA screening, of which 27 showed positive binding (data not shown). Sequencing of these clones identified nine distinct nanobodies—Nb25, Nb27, Nb55, Nb67, Nb70, Nb71, Nb72, Nb85, and Nb91—based on CDR3 hypervariable region sequences (**Figure 2A**). iELISA confirmed that all nine nanobodies specifically bound to NS4B but did not cross-react with BVDV NS5A, which was similarly expressed with a 6×His tag (**Figure 2B**). Among these, Nb25, Nb55, and Nb91 exhibited the highest binding affinity, with titers exceeding 1:2000 (**Figure 2C**). These three nanobodies, along with the non-binding control Nb5, were selected for subsequent study.

**Table 2:** Enrichment of NS4B-specific phages during three rounds of panning.

Round of panning	Phage input (PFU/Well)	Phage output (PFU/Well)	Recovery rate	Enrichment
1st round	1.4 × 10^12^	1.74 × 10^7^	1.24 × 10^-5^	4.8
2nd round	1.4 × 10^12^	5.6 × 10^10^	4 × 10^-2^	11.2
3rd round	1.4 × 10^12^	7.1 × 10^11^	5.07 × 10^-1^	5.46 × 10^2^

### 3.3 Inhibition of BVDV infection by intracellularly expressed nanobodies

**Figure 3:**
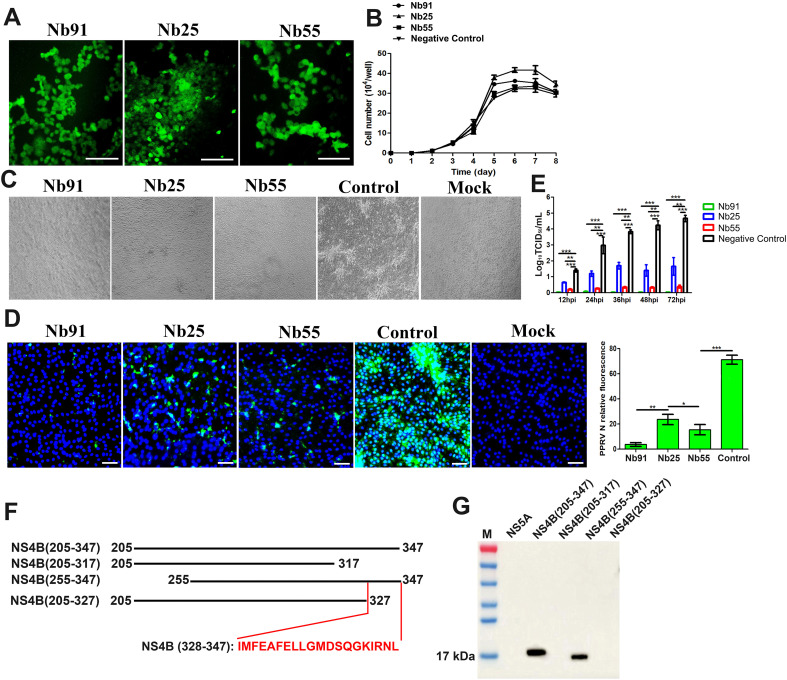
Intracellularly-expressed Nbs inhibits BVDV replication. Characterization of MDBK cell lines were performed by fluorescent microscopy **(A)**. Growth curves of MDBK-Nbs EGFP cell lines **(B)**. BVDV-induced CPEs were monitored under an inverted microscope **(C)**. BVDV replication levels were determined by using IFA **(D)** and the virus titers in the culture supernatants were measured based on the TCID_50_ value **(E)**. **(F)** Schematic diagram of different truncated C terminal of BVDV NS4B proteins. **(G)** The interaction of Nb91 with different truncated C terminal of NS4B proteins was determined using western blotting. The scale bar indicates 100 μm. *p*-values were calculated using Student's t test. An asterisk indicates a comparison with the indicated control. * *p* < 0.05; ** *p* < 0.01; *** *p* < 0.001.

To evaluate the antiviral effects of intracellular nanobodies, MDBK cell lines stably expressing Nb91, Nb25, or Nb55 were established (**Figure 3A**). A CCK-8 assay revealed that nanobody expression did not significantly affect cell viability compared to wild-type MDBK cells (**Figure 3B**). Upon infection with cytopathogenic (CP) BVDV at an MOI of 1, control MDBK cells exhibited pronounced cytopathic effects (CPE), which were markedly reduced in nanobody-expressing cells (**Figure 3C**). Immunofluorescence assays using an anti-BVDV N protein antibody demonstrated that intracellular expression of nanobodies caused an approximately 70%-90% reduction in virus positive staining (**Figure 3D**). TCID_50_ assays of supernatants showed an approximately 3 logs to 4 logs reduction in virus yield compared with the positive infected control cells at 72 hpi (**Figure 3E**). Notably, Nb91 expression nearly completely inhibited BVDV replication. Subsequently, to precisely determine the epitope recognized by Nb91, different truncated BVDV-N proteins were designed and expressed (**Figure 3F**). Using these fragments as antigens, western blotting results showed that Nb91 reacted with fragments spanning amino acids 205–347 and 255-347, but not 205–317 and 205-327, suggesting that the epitope was located within amino acids 328-347 (**Figure 3G**).

### 3.4 Expression and purification of TAT-fused nanobodies

**Figure 4:**
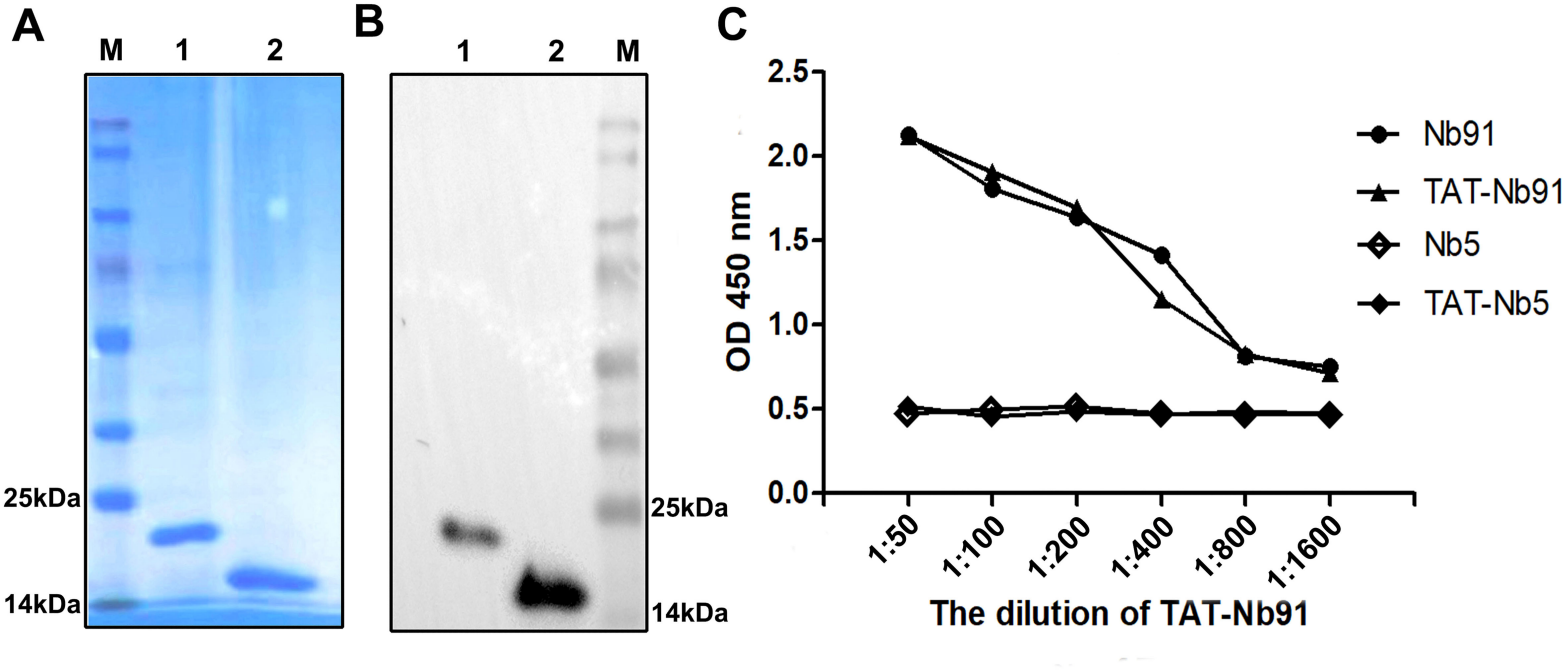
The expression, purification, and identification of TAT-Nb91 recombinant protein. Analysis of purified and refolded nanobodies by SDS-PAGE **(A)** and Western blotting **(B).** The predicted sizes of the His-tagged Nb91 are 15 kDa without TAT and 19 kDa with the TAT leader peptide. M, protein marker; lane 1, TAT-Nb91; lane 2, Nb91. Determination of the binding activity of TAT-Nb91 and Nb91 to NS4B by iELISA **(C)**. TAT-Nb5 was used as a negative control.

To enhance cellular delivery, Nb91 was fused to a TAT peptide. The fusion protein TAT-Nb91 and unfused Nb91 were expressed in *E. coli*, purified under denaturing conditions using Ni-NTA resin, refolded, and dialyzed. SDS-PAGE and Western blot analysis confirmed the expected sizes of approximately 19 kDa for TAT-Nb91 and 15 kDa for Nb91 (**Figures 4A, B**). iELISA showed that both forms specifically bound to NS4B, with no significant difference in reactivity (**Figure 4C**). TAT-Nb5 served as a negative control.

### 3.5 Cellular internalization of TAT-Nb91

**Figure 5:**
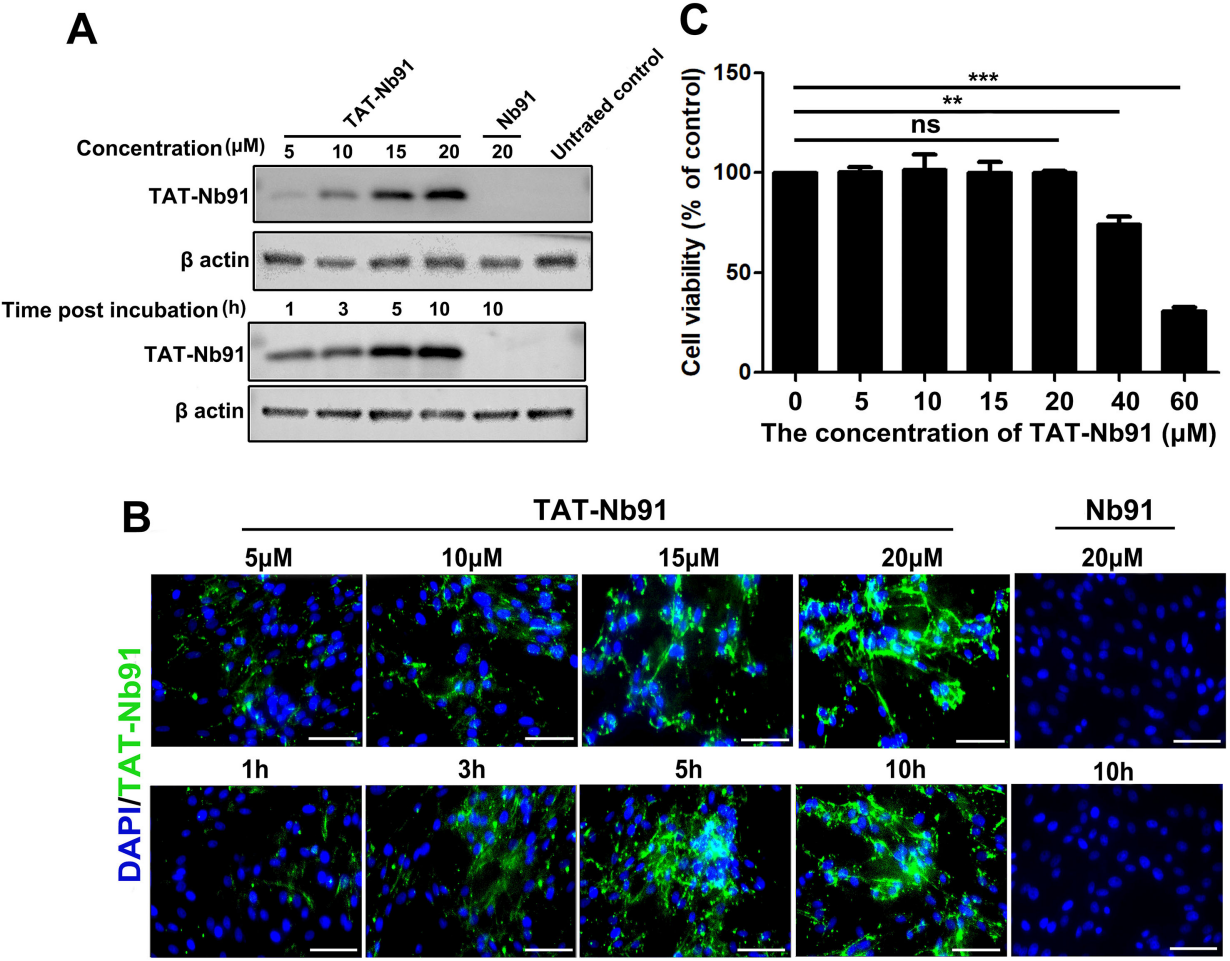
Cellular uptake of TAT-Nb91 into MDBK cells. Cellular uptake of TAT-Nb91 and Nb91 at indicated concentration for 5 h or at 20 μM for indicated time were examined by western blot **(A)** and IFA assay **(B)**. **(C)** Detection of TAT-Nb91 toxicity using CCK-8 kits. The scale bar indicates 100 μm. *p*-values were calculated using Student's t test. An asterisk indicates a comparison with the indicated control. ** *p* < 0.01; *** *p* < 0.001.

The cellular uptake of TAT-Nb91 was evaluated in MDBK cells treated with various concentrations of the nanobodies for different time. Western blot and immunofluorescence analysis revealed that TAT-Nb91 entered cells in a dose- and time-dependent manner, whereas unfused Nb91 showed no detectable internalization (**Figures 5A, B**). A CCK-8 assay indicated that TAT-Nb91 was not cytotoxic at concentrations up to 20 μM, though viability declined at 40 μM (**Figure 5C**).

### 3.6 Inhibition of BVDV replication by TAT-Nb91

**Figure 6:**
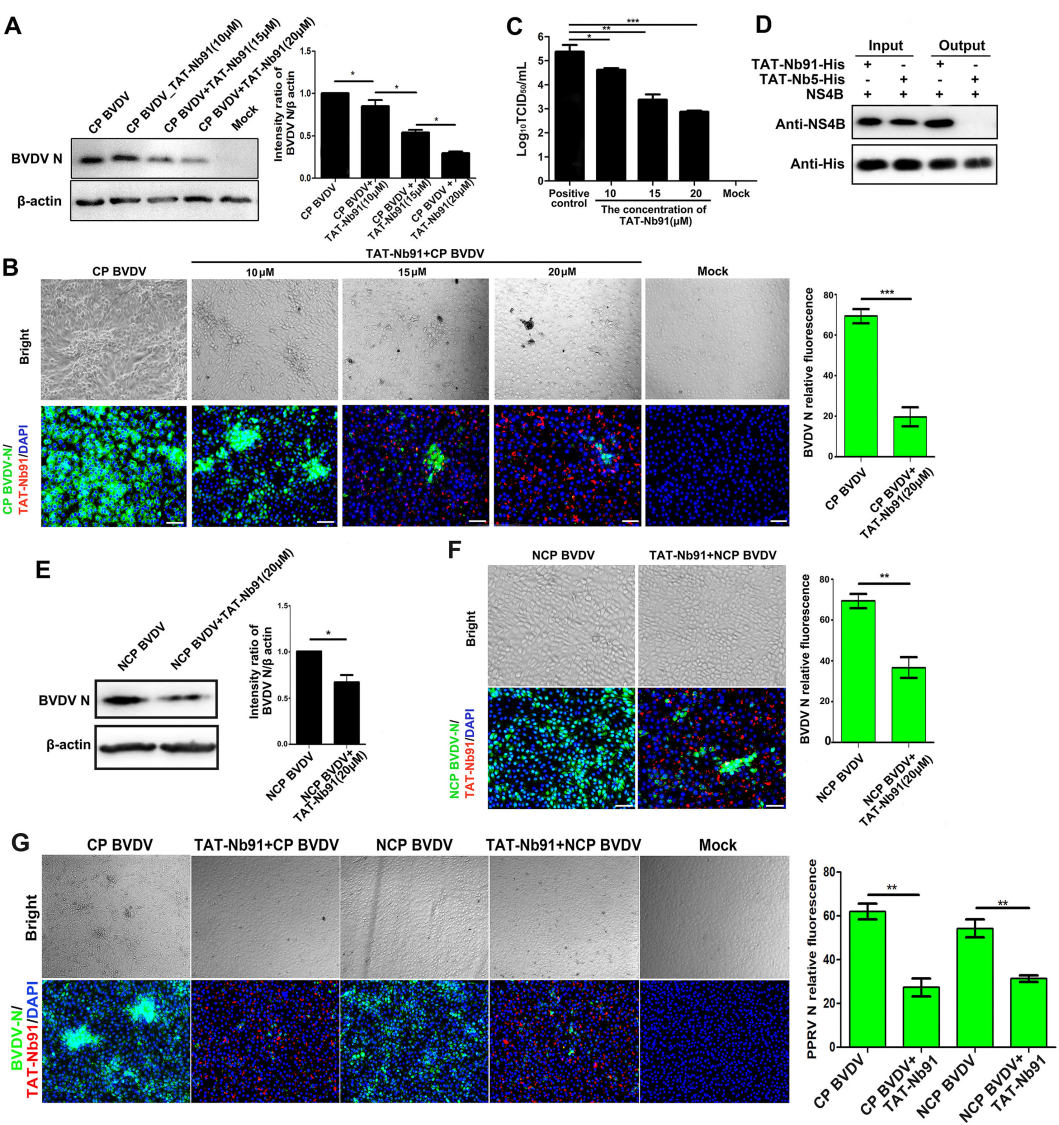
Inhibition of BVDV replication by TAT-Nb91 in MDBK cells. MDBK cells were infected with CP BVDV at an MOI of 1 for 1 h, and then the cell culture media were replaced with fresh DMEM containing 3% FBS and TAT-Nb91 at the indicated concentrations. TAT-Nb91 and BVDV N were detected at 24 hpi by Western blotting **(A)** and IFA **(B)** using anti-His MAb and mouse anti-BVDV N polyantibody, respectively. Progeny virus released in the cell medium was measured by TCID_50_ at 24 hpi **(C)**. MDBK cells were infected with CP BVDV at an MOI of 1 for 48 h, and NS4B was pulled down by TAT-Nb91-His. The bound proteins were detected by Western blotting using mouse anti-His antibody or mouse anti-BS4B antiserum **(D)**. MDBK cells were infected with NCP BVDV at an MOI of 1 for 1 h, and virus replication levels were determined by Western blot **(E)** and IFA **(F).** Inhibition of BVDV replication by TAT-Nb91 in Bend cells **(G)**. The scale bar indicates 100 μm.

To evaluate the ability of TAT-Nb91 to inhibit BVDV infection, MDBK cells infected with CP BVDV (strain HJ-1, MOI = 1) were treated with TAT-Nb91 at various concentrations. Western blot and IFA analysis showed a dose-dependent reduction in BVDV N protein expression (**Figures 6A, B**), and TCID_50_ assays confirmed a decrease in viral titers in a dose-dependent manner (**Figure 6C**). In particularly, compared with infected control cells, BVDV N expression levels and progeny virus in cells treated with TAT-Nb91 at 20 μM significantly decreased by approximately 70% and 2.5 logs, respectively. To validate the binding ability of TAT-Nb91 to the native NS4B produced in virus infection, pulldown assays were performed. As expected, NS4B was pulled down by TAT-Nb91-His but not by the control nanobody, TAT-Nb5-His, suggesting that TAT-Nb91 maintained the antigen binding ability of the nanobody (**Figure 6D**). Moreover, similar experiments using the noncytopathogenic (NCP) strain New York 1 demonstrated that TAT-Nb91 also suppressed NCP BVDV replication by approximately 40% (**Figures 6E, F**), although less antiviral effect than CP BVDV. Since TAT-Nb91 exerted significant antiviral activity in both CP and NCP biotype of BVDV-infected MDBK cells, we further investigated whether TAT-Nb91 could suppress BVDV replication in Bend cells, the primary targets of BVDV infection *in vivo*. Bend cells were treated with TAT-Nb91 at 20 μM. IFA analysis showed that TAT-Nb91 treatment caused both CP and NCP biotype of BVDV N expression decreased by 60% and 40%, respectively (**Figure 6G**).

## 4 Discussion

Intrabodies represent a promising therapeutic agent with considerable potential for the treatment of viral infections, capable of targeting both early and late stages of the viral life cycle (20, 21). Nanobodies (Nbs) offer several advantageous properties, including high specificity, strong binding affinity, notable stability, solubility, and minimal size (22, 23). Despite these benefits, their inability to passively cross cell membranes limit their ability against intracellular targets. CPPs have emerged as effective vehicles for the intracellular delivery of therapeutic proteins. Previous studies have demonstrated that CPP-conjugated nanobodies can traverse the plasma membrane and inhibit the replication of diverse viruses (24–26), underscoring their potential as antiviral agents.

Bovine viral diarrhea still remains a major challenge in the global cattle industry due to the genetic diversity of BVDV and the absence of effective treatments (1). In this study, we isolated a BVDV NS4B-specific nanobody, Nb91, and enhanced its cellular delivery by fusion with the TAT peptide. Importantly, TAT-Nb91 significantly inhibited the replication of both cytopathic (CP) and noncytopathic (NCP) BVDV strains in MDBK cells and Bend cells.

RNA viruses exhibit high genetic variability, particularly in neutralizing epitopes of structural proteins under immune selection pressure. In contrast, nonstructural proteins such as BVDV NS4B, which are involved in cytoplasmic replication complexes, are generally more conserved (8). NS4B, essential for viral replication due to its hydrophobic properties, is an attractive target for antiviral development (6). It facilitates the formation of the viral replication complex and contributes to viral pathogenesis and autophagy induction (6). Although a nanobody targeting NS5B has previously been shown to inhibit BVDV replication (15), the antiviral potential of NS4B-specific nanobodies, particularly when delivered via CPPs, remains underexplored.

In this study, we performed phage display to isolate NS4B-specific nanobodies. Nine distinct Nbs were identified, among which three high-affinity binders, including Nb25, Nb55, and Nb91, were selected for functional evaluation. Interestingly, despite strong binding affinities *in vitro*, these nanobodies exhibited different antiviral ability. These findings suggest that the high affinity of nanobodies does not necessarily translate to direct antiviral activity. Moreover, Nb91 demonstrated superior inhibition of viral replication compared to Nb25 and Nb55, suggesting it may target a critical functional epitope of NS4B, potentially disrupting its hydrophobic activity required for virus replication. Subsequently, it was determined that the conserved region of amino acids 328–347 in NS4B protein was the essential domain for binding to Nb91. NS4B protein is relatively well conserved compared with other BVDV proteins, and the amino acid sequence between CP and NCP biotype BVDV NS4B was 99%-100% (1). These may explain the inhibition of NCP BVDV replication by TAT-Nb91. As nanobodies unique advantages over conventional antibodies, they hold significant promise for clinical applications (27). A major challenge, however, lies in the efficient intracellular delivery of nanobody proteins. While gene therapy approaches represent one strategy (28), CPP fusion offers a direct protein delivery alternative. The TAT peptide has been shown to mediate dose- and time-dependent cellular uptake of various cargo proteins (29), a finding corroborated in our study with TAT-Nb91 in MDBK cells (**Figure 5**).

The subcellular localization of TAT-fused proteins remains a subject of debate. While some studies report nuclear accumulation of TAT-GFP, others observe cytoplasmic localization (30–32). Our results align with the latter, showing predominant cytoplasmic distribution of TAT-Nb91 (33), which may be influenced by cell type, culture conditions, or the nature of the cargo protein. This localization is functionally relevant as Nb91 likely targets cytoplasmic NS4B. The precise antiviral mechanism of TAT-Nb91 warrants further investigation. Notably, TAT-Nb91 exhibited no cytotoxicity at concentrations up to 20 μM, but viability decreased significantly at 40 μM, highlighting the importance of dosage control for therapeutic application.

Evaluation of antiviral activity revealed that TAT-Nb91 inhibits both CP and NCP BVDV strains in MDBK cells and Bend cells, although with varying efficacy. The stronger suppression of CP BVDV may be attributable to biotype-specific differences in NS4B. Although NS4B is relatively conserved, a single point mutation (Y2441C) is known to modulate viral cytopathogenicity (34). Such variations may influence Nb91 binding and antiviral potency against NCP strains, a hypothesis requiring further validation. However, it should be noted that the antiviral effect of nanobody was controversial *in vitro* and *in vivo*. The inhibition efficiency of TAT-Nb91 on BVDV replication and potential toxicity of TAT-Nb91 *in vivo* remain to be investigated.

## 5 Conclusion

In summary, this study demonstrates that the CPP-fused nanobody TAT-Nb91, produced in a prokaryotic expression system, efficiently enters MDBK cells in a dose- and time-dependent manner and inhibits the replication of both CP and NCP BVDV strains. Importantly, the region of amino acids 328–347 in the C-terminal part of NS4B protein was determined to be the key amino acid for interacting with Nb91. These findings not only provide insights into the molecular basis of BVDV NS4B protein as a key factor for viral replication but also highlight a novel therapeutic target for the development of anti-BVDV replication drugs. Ongoing studies are focused on elucidating the molecular mechanism underlying Nb91-mediated suppression of BVDV replication and evaluating the *in vivo* efficacy of TAT-Nb91.

## Data Availability

The datasets presented in this study can be found in online repositories. The names of the repository/repositories and accession number(s) can be found in the article/supplementary material.
